# Sequence and structural determinants of human APOBEC3H deaminase and anti-HIV-1 activities

**DOI:** 10.1186/s12977-014-0130-8

**Published:** 2015-01-22

**Authors:** Mithun Mitra, Dustin Singer, Yu Mano, Jozef Hritz, Gabriel Nam, Robert J Gorelick, In-Ja L Byeon, Angela M Gronenborn, Yasumasa Iwatani, Judith G Levin

**Affiliations:** Section on Viral Gene Regulation, Program in Genomics of Differentiation, Eunice Kennedy Shriver National Institute of Child Health and Human Development, National Institutes of Health, Bethesda, MD 20892-2780 USA; Clinical Research Center, National Hospital Organization Nagoya Medical Center, Nagoya, Aichi 460-0001 Japan; Department of Structural Biology, University of Pittsburgh Medical School, Pittsburgh, PA 15261 USA; Pittsburgh Center for HIV Protein Interactions, University of Pittsburgh Medical School, Pittsburgh, PA 15261 USA; AIDS and Cancer Virus Program, Leidos Biomedical Research, Inc., Frederick National Laboratory for Cancer Research, Frederick, MD 21702-1201 USA; Department of Molecular, Cell and Developmental Biology, University of California, Los Angeles, CA 90095 USA; Department of Structural Biology, CEITEC, Masaryk University, Kamenice 5, 625 00 Brno, Czech Republic

**Keywords:** HIV-1, APOBEC3H, Homology model, Deaminase activity, Antiviral activity, Deaminase-independent restriction, Reverse transcription

## Abstract

**Background:**

Human APOBEC3H (A3H) belongs to the A3 family of host restriction factors, which are cytidine deaminases that catalyze conversion of deoxycytidine to deoxyuridine in single-stranded DNA. A3 proteins contain either one (A3A, A3C, A3H) or two (A3B, A3D, A3F, A3G) Zn-binding domains. A3H has seven haplotypes (I-VII) that exhibit diverse biological phenotypes and geographical distribution in the human population. Its single Zn-coordinating deaminase domain belongs to a phylogenetic cluster (Z3) that is different from the Z1- and Z2-type domains in other human A3 proteins. A3H HapII, unlike A3A or A3C, has potent activity against HIV-1. Here, we sought to identify the determinants of A3H HapII deaminase and antiviral activities, using site-directed sequence- and structure-guided mutagenesis together with cell-based, biochemical, and HIV-1 infectivity assays.

**Results:**

We have constructed a homology model of A3H HapII, which is similar to the known structures of other A3 proteins. The model revealed a large cluster of basic residues (not present in A3A or A3C) that are likely to be involved in nucleic acid binding. Indeed, RNase A pretreatment of 293T cell lysates expressing A3H was shown to be required for detection of deaminase activity, indicating that interaction with cellular RNAs inhibits A3H catalytic function. Similar observations have been made with A3G. Analysis of A3H deaminase substrate specificity demonstrated that a 5′ T adjacent to the catalytic C is preferred. Changing the putative nucleic acid binding residues identified by the model resulted in reduction or abrogation of enzymatic activity, while substituting Z3-specific residues in A3H to the corresponding residues in other A3 proteins did not affect enzyme function. As shown for A3G and A3F, some A3H mutants were defective in catalysis, but retained antiviral activity against HIV-1*vif* (−) virions. Furthermore, endogenous reverse transcription assays demonstrated that the E56A catalytic mutant inhibits HIV-1 DNA synthesis, although not as efficiently as wild type.

**Conclusions:**

The molecular and biological activities of A3H are more similar to those of the double-domain A3 proteins than to those of A3A or A3C. Importantly, A3H appears to use both deaminase-dependent and -independent mechanisms to target reverse transcription and restrict HIV-1 replication.

**Electronic supplementary material:**

The online version of this article (doi:10.1186/s12977-014-0130-8) contains supplementary material, which is available to authorized users.

## Background

The human APOBEC3 (A3) family consists of seven cytidine deaminases that catalyze the conversion of deoxycytidine (dC) to deoxyuridine (dU) in single-stranded (ss)DNA, thereby inducing G-to-A hypermutation in double-stranded DNA [1-5]. A3 proteins play an important role in the innate immune defense system by inhibiting a broad range of exogenous viruses such as human immunodeficiency virus type 1 (HIV-1) (reviewed in refs. [6-13]), human T-lymphotropic virus type 1 (HTLV-1) [14,15], and hepatitis B virus (HBV) [16,17] as well as endogenous retrotransposons such as LINE-1 and Alu elements (reviewed in refs. [7,18]). These proteins contain either one (A3A, A3C, and A3H) or two (A3B, A3D (formerly known as A3D/E), A3F, and A3G) Zn-binding domains with the conserved motif **H**X_1_**E**X_23-24_**C**X_2-4_**C** (X is any amino acid) [19] (reviewed in refs. [20,21]). The histidine and two cysteines coordinate a Zn ion, while the glutamic acid residue is thought to act as a proton shuttle during catalysis [6,22]. Based on phylogenetic analysis, the Zn-binding domains were further classified into the following groups: Z1 (A3A and C-terminal domains (CTD) of A3B and A3G), Z2 (A3C, N-terminal domains (NTD) of A3B and A3G, and both NTD and CTD of A3D and A3F), and Z3 (A3H) [23,24].

A3H is the most divergent member of the A3 family and has a single Zn-binding domain that belongs to the unique Z3 group [24,25]. The A3H message undergoes alternative splicing to generate variants containing distinct C-terminal regions [26,27]. Furthermore, unlike other *A3* genes, *A3H* is present in the human population as different haplotypes containing functional polymorphisms. At present, seven haplotypes of A3H (Hap I-VII) have been identified that differ in their antiviral activities: only Hap II, Hap V, and Hap VII are stably expressed and are able to restrict Vif-deficient HIV-1 [26-29]. Interestingly, the distribution of A3H haplotypes in the human population is correlated with geographical location [26,29]. For example, a higher frequency of HapII is present in Africa, compared to Europe and Asia, possibly due to a greater selection pressure against pathogens endogenous to that region [26].

HIV-1 Vif, which counteracts antiviral activity by promoting proteasomal degradation of A3C, A3D, A3F, and A3G, exhibits different degrees of potency against the individual A3H haplotypes [12,29-32]. In cell-based assays, the sensitivity of antiviral A3H HapII towards Vif was shown to be dependent upon the Vif subtype [33,34] and a remarkable study involving recently infected HIV-1 patients revealed adaptive changes in viral Vif sequences that were attributed to the presence of the different antiviral A3H haplotypes [32]. These observations provide strong evidence for a significant role of A3H as an antiviral defense protein.

A3 proteins deaminate dC residues in a sequence-specific manner. For example, A3A exhibits a greater preference for the dC in the center of a T**C**A target [35-42], while A3G specifically deaminates the dC in a CC**C** motif [21,43-45]. Evaluation of the structural basis of the sequence specificity suggested that it is determined by the architecture of the active site and surrounding amino acids, in particular, residues in loop 7 [41-46] (reviewed in refs. [21,47]). Although, A3H is known to deaminate dC in T**C** motifs of HIV-1 minus-strand DNA [27,48], a detailed investigation of the nucleotide context immediately 5′ and 3′ of the dC on sequence-specific deamination has not been reported. In addition, the amino acid residues that are important for A3H deaminase activity and the role of structure in dictating biological function have not been investigated.

In the present study, we focus on the biochemical and structural determinants of A3H HapII (to be referred to as “A3H”) deaminase and antiviral activities, using site-directed and structure-guided mutagenesis. We have constructed a homology model of A3H and find that the A3H structure, as expected, is similar to the known structures of A3A [41], A3C [49], A3G-CTD [43,50-53], and A3F-CTD [54,55], with differences mainly in flexible loop regions. Our model resembles the ones generated by (i) MODELLER [56], based on the A2 and A3G-CTD structures [57], and (ii) the automated structure-homology-modeling server, SWISS-MODEL, using the A2 structure [13], although details may be different. Interestingly, our model also reveals a large cluster of basic residues, which is not present in other A3 deaminase-active domains, and is consistent with the observation that deaminase activity in cell-free extracts is detected only after removal of RNA by treatment with RNase A. In addition, we have evaluated the deaminase and antiviral activities of a series of A3H mutants. Although these activities can be correlated in most cases, a significant number of mutants lacking enzymatic activity are still able to inhibit HIV-1 replication, albeit at a lower efficiency than wild type (WT). This result raises the possibility that A3H restricts HIV-1 by catalytic-dependent and -independent mechanisms. Indeed, assays of endogenous reverse transcription (ERT) support this hypothesis. Taken together, our findings provide new insights into the role of A3H as a naturally occurring human restriction factor and should contribute to continuing efforts to combat HIV infection in the African human population.

## Results

### Sequence- and structure-based design of A3H mutants

In this work, we set out to investigate the determinants of A3H cytidine deaminase and antiviral activities, using a mutagenic approach. Given A3H's unique Z3-type Zn-binding domain [24], we initially carried out a sequence comparison of the Z3 domain of A3H with the Z1 and Z2 domains of other A3 proteins to identify conserved and distinct regions in A3H (Additional file 1: Figure S1). Sequence identities range from 28-43% and the Z3 domain shares the greatest identity with the Z2 domains of A3C and A3F-CTD and the least with A3D-NTD. The sequence alignment also identified four residues unique to the Z3 domain: T81, L102, S109, and V135, which are replaced by S, V, A, and I in other Z domains.

**Figure 1:**
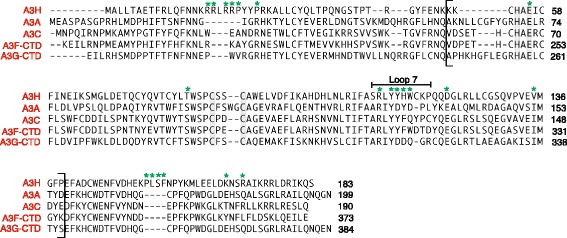
**Sequence alignment of A3H HapII (residues 1 to 183) and four A3 deaminase-active proteins whose structures have been solved: A3A (1 to 199) [** 41 **], A3C (1 to 190) [** 49 **], A3F-CTD (185–373) [** 54 **,** 55 **], and A3G-CTD (191–384) [** 53 **] (also see refs. [** 43 **,** 50 **-** 52 **]).** The A3H residues mutated in this study are highlighted with green asterisks and the active site residues are highlighted in blue. The region inside the square brackets represents the Z domain sequences for all of the proteins. The amino acids that comprise A3H loop 7 are also shown. The sequence alignment was generated using Lasergene software (DNASTAR, Inc., Madison, WI, USA).

A more extensive sequence alignment was performed by comparing the residues in the complete A3H protein with the sequences of A3 proteins whose three-dimensional structures have been solved at high resolution, i.e., A3A [41], A3C [49], A3F-CTD [54,55] and A3G-CTD [43,50-53] (Figure 1). The overall sequence identity between A3H and each of these proteins is very similar and ranges from 35 to 38% (Table 1). The Z domains (bracketed) include the loop 7 residues, a region that is involved in deaminase substrate specificity [41-46] (reviewed in [21,47]). Interestingly, loop 7 sequences for different A3 proteins display alternative arrangements of polar and non-polar residues. For example, A3H contains a stretch of aromatic residues (YYHW, 112–115), while in A3G-CTD, the corresponding residues are polar (YDDQ, 315–318). In addition, a unique stretch of residues was noted outside the Z domains, namely 154–157 (PLSF), which is absent in the other A3 proteins.

**Table 1:** **Sequence comparison of A3H with other deaminase-active A3 proteins and theoretical isoelectric points (pI)**

	**Zinc-binding domain (Z) type**	**% Sequence identity to A3H** ^**a**^	**Theoretical pI** ^**b**^
**A3A (1–199)**	Z1	36	6.3
**A3C (1–190)**	Z2	38	7.5
**A3F-CD2 (185–373)**	Z2	36	5.0
**A3G-CD2 (191–384)**	Z1	35	6.2
**A3H HapII (1–183)**	Z3	100	8.9

^a^Sequence identity analysis was performed using Lasergene software (DNASTAR, Inc.).

^b^The theoretical pI values were calculated using the Protparam online web-based program (http://web.expasy.org/compute_pi/).

**Figure 2:**
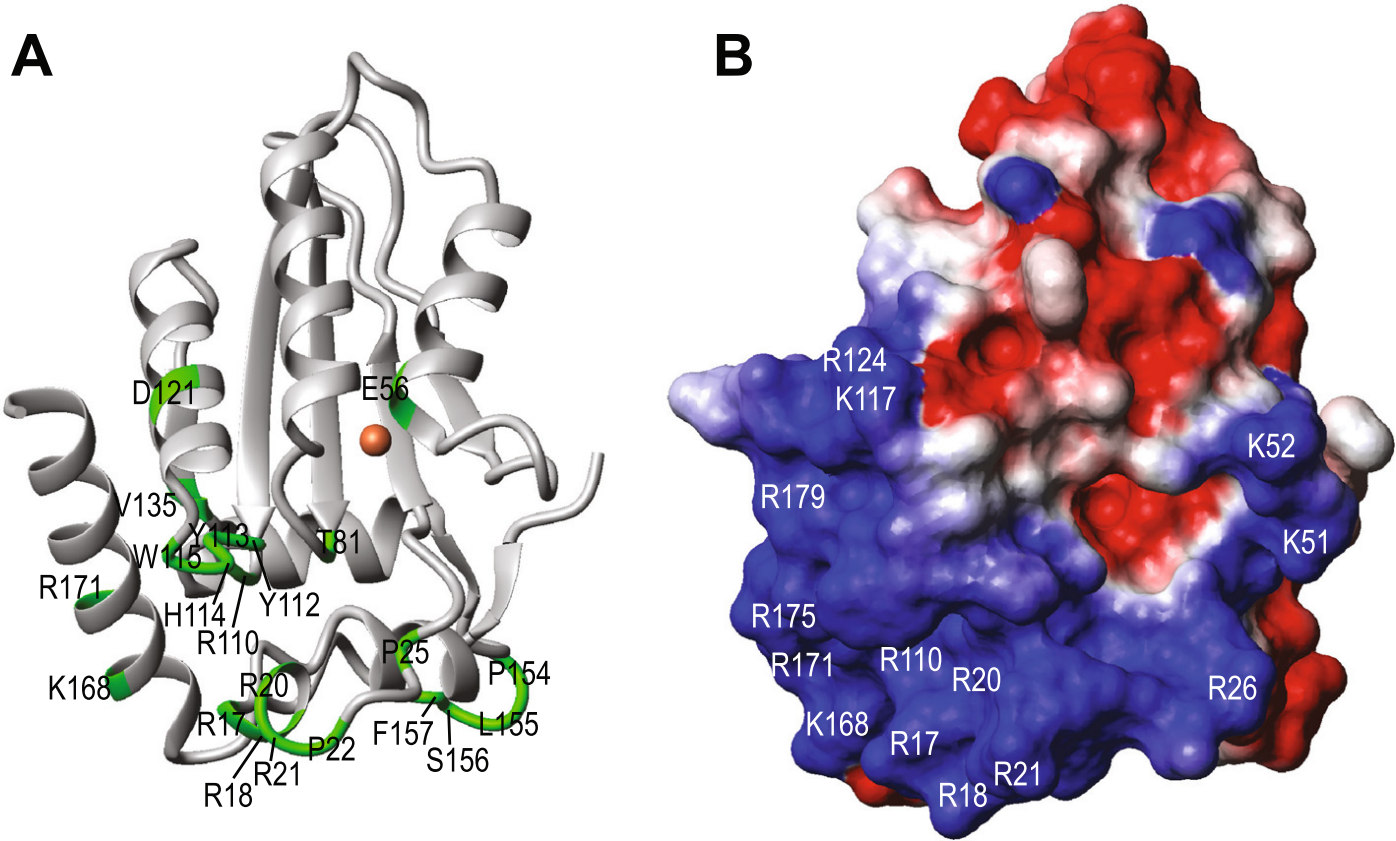
**A3H model structure. (A)** Ribbon representation of the A3H model showing the positions of residues mutated in this study in green. The Zn ion is colored in brown. **(B)** Electrostatic map of the A3H model depicting regions with positive (blue) and negative (red) electrostatic potentials. Basic residues are shown in white.

To probe the role of A3H residues in enzymatic activity in relation to their location in the structure, we constructed a homology model based on the X-ray structure of A3G-CTD [53] (Figure 2A). Comparison of our A3H model with A3G-CTD (PDB: 3IR2) [53], A3A (PDB: 2 M65) [41], A3C (PDB: 3 VOW) [49], and A3F-CTD (PDB: 4IOU) [54] showed that the A3H model is similar to the other A3 structures, which have r.m.s.d. values ranging from 3.1 to 3.5 Å. The major differences between the various A3 structures occur in the loops, as suggested previously [41] (Additional file 2: Figure S2).

Comparisons of the electrostatic surface features of A3H (Figure 2B, Additional file 3: Figure S3) and other A3 proteins (A3G-CTD, A3A, A3C, and A3F-CTD) (Additional file 3: Figure S3) reveal a striking difference in the clustering of basic residues (blue regions), consistent with the fact that A3H is highly basic (theoretical pI, 8.9) compared to the other single-domain A3 proteins A3A (pI, 6.3) and A3C (pI, 7.5) (Table 1). Indeed, the basic character of A3H is more similar to the positively charged N-terminal domains (NTDs) of the double-domain proteins, A3F (residues 1–180; pI, 8.6) and A3G (residues 1–185; pI, 9.4). The NTDs of A3F and A3G play an important role in binding viral RNA that is packaged; however, they are enzymatically inactive [58-61]. Since A3H is a single-domain protein, it is likely that the basic residues serve a dual function, i.e., binding both viral and/or cellular RNA as well as interacting with the ssDNA substrate for deamination.

### RNA inhibits the deaminase activity of A3H

**Figure 3:**
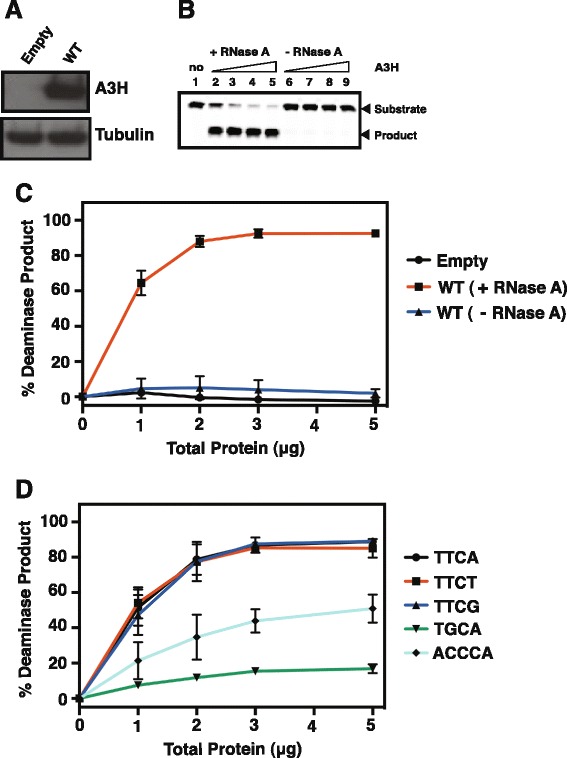
**A3H deaminase activity dependence on RNase A treatment of extracts and substrate specificity. (A)** Western blot analysis showing the WT A3H protein levels in 293T cells. Transfection of the empty vector (no A3H) served as a negative control and showed that 293T cells do not contain detectable levels of endogenous A3H. The tubulin loading control is also shown. **(B)** Representative gel illustrating assay of WT A3H deaminase activity in a cell extract using a 40-nt TT**C**A-containing oligonucleotide substrate. The oligonucleotide was incubated with increasing amounts of A3H extract in the presence and absence of RNase A. The positions of the substrate (40 nt) and the deamination product are indicated by arrows to the right of the gel. Lane 1, empty vector control; lanes 2 and 6, lanes 3 and 7, lanes 4 and 8, and lanes 5 and 9 represent reactions containing 1 μg, 2 μg, 3 μg, and 5 μg of total protein, respectively. **(C)** The percent (%) deamination product was calculated as described in Methods and was plotted against the amounts of total protein. **(D)** Deaminase assay using WT A3H extract and 40-nt oligonucelotides containing the following deaminase motifs: TT**C**A, TT**C**T, TT**C**G, TG**C**A, and ACC**C**A. The data were analyzed and plotted as described in **(C)**.

Deaminase activity of A3H variants was tested in 293T cell lysates using a TT**C**A-containing 40-nt oligonucleotide as described in Methods; protein expression was monitored by Western blot analysis, using an A3H-specific antibody probe (Figure 3A). We selected the TT**C**A motif, since analysis of HIV-1 viral DNA showed that the dC residue in T**C** motifs was preferentially deaminated by A3H [27,32,48,62]. Surprisingly, we observed only trace amounts of deaminase activity in (WT) A3H cell lysates (Figure 3B and C). However, activity was clearly detectable after treating these lysates with RNase A, while no effect was seen with cells transfected with the empty vector control. These results suggest that A3H is bound to RNA present inside the cell and that this interaction with RNA inhibits A3H deaminase activity, presumably in a competitive manner. Interestingly, a similar inhibitory effect on enzyme function was also observed with A3G [7,63].

### Deaminase target specificity of A3H

Until now, detailed analysis of A3H deaminase specificity has not been reported. We therefore performed deaminase assays using oligonucleotides containing different motifs: TT**C**A, TT**C**T, TT**C**G, TG**C**A, and ACC**C**A, where either a purine (G) or a pyrimidine (T or C) is present at the position immediately 5’ of the target dC residue. As shown in Figure 3D, the presence of a 5′ T (e.g., TT**C**A, TT**C**T, and TT**C**G) yielded the highest amounts of deamination product (~90% substrate conversion with 5 μg of total protein after incubation for 1 h at 37°C), while a 5′ G, as in TG**C**A, was poorly tolerated (~17%). The rank order of deamination efficiency of the substrates is: TT**C**A ~ TT**C**T ~ TT**C**G > ACC**C**A > TG**C**A. These data suggest a less stringent requirement for the 3′ position, where either a purine or a pyrimidine is tolerated, since similar levels of deamination product were formed with substrates containing TT**C**A, TT**C**T, and TT**C**G motifs. (Note that some difference in the deamination efficiency of substrates with TT**C**A, TT**C**T, and TT**C**G motifs might occur if less than 1 μg of lysate were added to the reaction.)

### Identification of A3H residues important for deaminase activity

**Figure 4:**
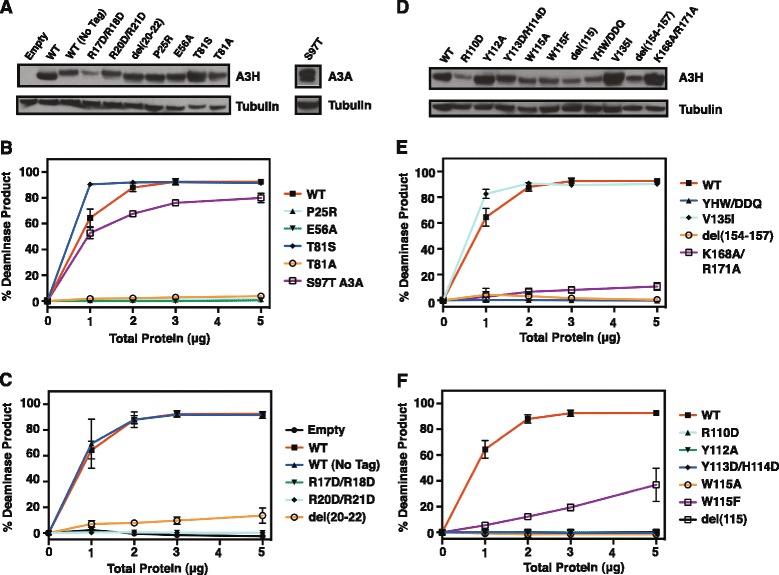
**Deaminase activities of A3H WT and mutants. (A) and (D)** Western blot analysis showing the amounts of A3H WT and mutants and A3A S97T in 293T cells. **(B, C, E, F)** Deaminase assays using 293T cell extracts expressing A3H WT and mutants and A3A S97T. The deaminase assays were performed as described in Methods.

**Figure 5:**
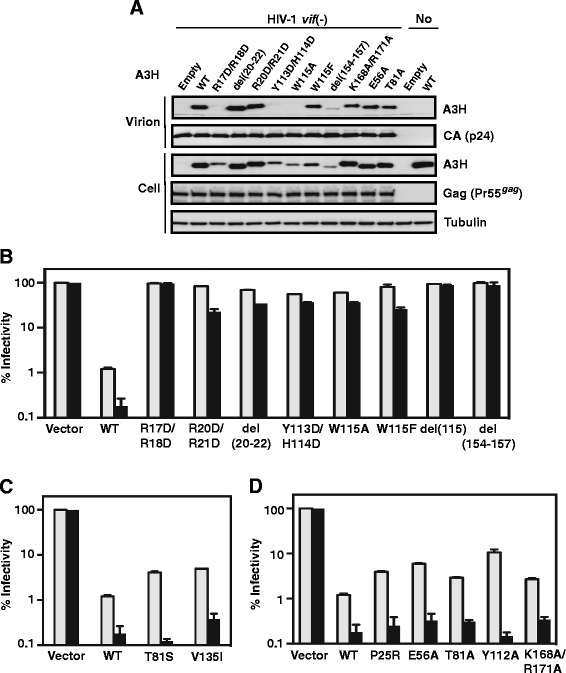
**Antiviral activities of A3H WT and mutants. (A)** Western blot analysis showing the amounts of A3H WT and mutants in HIV-1*vif* (−) virions and 293T cells. 293T cells were transfected with HIV-1*vif* (−) and A3H WT or mutant plasmids at a 1:1 ratio (1 μg each for determination of expression in cell extracts and 8 μg each for determination of A3H in viral lysates). Cell extracts (10 μg of total protein) as well as viral lysates (8 μl of viral pellet resuspended in 200 μl of loading buffer) were subjected to Western blot analysis. Viral lysates were probed with antibodies to the N-terminal FLAG tag of A3H and HIV-1 CA; cell extracts were probed with antibodies to the N-terminal FLAG tag of A3H, HIV-1 Gag (Pr55^*gag*^), and tubulin. Controls: Left side, EMPTY refers to HIV-1*vif* (−) and empty vector (pTR600); Right side, EMPTY refers to empty vector alone; WT, refers to A3H plasmid DNA alone. **(B-D)** Antiviral activity was determined as described in Methods. The gray and black bars respresent transfection with 0.1 μg or 1 μg of the indicated A3H plasmid, respectively.

The sequence-structure analysis (Figures 1 and 2) provided a rationale for mutagenesis of A3H residues that could potentially play a role in catalytic activity. Deaminase and HIV-1 infectivity assays of WT and mutant constructs were performed in parallel to evaluate enzymatic (Figure 4) and antiviral (Figure 5) activities. The data in Figure 4A and D are arranged according to residue position along the polypeptide chain from the N- to C-terminus. As expected, the active site mutant E56A (negative control) did not display any deaminase activity (Figure 4B).

In our initial screen, we focused on residues that are Z3-specific (Additional file 1: Figure S1), in order to probe whether these residues were selected throughout evolution to ensure A3H enzymatic activity. We chose two examples from this group, T81 and V135, which correspond to S and I, respectively, in Z1 and Z2 Zn-binding domains (Figure 1). Mutants with these Z1-Z2 substitutions were expressed at levels comparable to or higher than WT (Figure 4A and D). Interestingly, changing T81 to S did not reduce deaminase activity, whereas the T81 to A mutation completely abolished activity (Figure 4B). Note that the mutant protein T81A was expressed at levels comparable to WT (Figure 4A). Since both threonine and serine possess a hydroxyl group, while alanine does not, this may suggest that the side chain of T81 is involved in a polar interaction. Changing the analogous residue S97 in A3A (Z1 type) to T did not affect deaminase activity (Figure 4B), consistent with retention of the important hydroxyl group. Similarly, mutating V135 to I also represents a conservative change and not surprisingly, there was no effect on enzymatic activity (Figure 4E).

In contrast, deletion of the unique stretch of amino acids, PLSF (aa 154–157), which contains three bulky hydrophobic residues, proline, leucine, and phenylalanine, reduced enzymatic activity to background level (Figure 4E). This suggests that a major conformational change was introduced by the deletion, causing an overall structural defect. In turn, this would lead to destabilization of the protein, which could explain the low protein expression level of this mutant (Figure 4D) and inability to be packaged efficiently (see below) (Figure 5A). Changing another unique A3H residue, P25 to R, abrogated A3H deaminase activity (Figure 4B), although its expression level was normal (Figure 4A).

Basic residues in A3 proteins are often involved in specific and non-specific nucleic acid interactions [21,47,64]. To examine the role of these residues in A3H deaminase activity (Figure 4C and F) and to determine whether the location of these residues in the A3H model structure is related to their function, we focused on residues in the “basic patch” (Figure 2B): R17, R18, R20, R21, R110, K168, and R171. Note that R18, R20, and R21 are not present in other A3 proteins (Figure 1). We constructed a single mutant with an R → D change (R110D) as well as double mutants R17D/R18D, R20D/R21D, and K168A/R171A. With the exception of R17D/R18D and R110D, the mutant proteins were efficiently expressed (Figure 4A and D), but almost all lacked deaminase activity (Figure 4C, 4E, and F), even at high amounts of total protein. Two mutants, del(20–22) (Figure 4C) and K168A/R171A (Figure 4E), displayed greatly reduced, but measurable activity (~15% and ~11% product, respectively, at 5 μg total protein). These results suggest that positive charges are necessary for binding of A3H to the ssDNA substrates and that the introduction of a single negative charge in the basic patch disrupts the favorable charge-charge interaction. The change to the non-polar alanine or deletion of residues 20–22, which are present only in A3H as a unique insertion, is less detrimental. Taking all of the above data together, it appears likely that these basic residues form part of the A3H nucleic acid binding interface.

Another region of interest, loop 7 (Figure 1), which in other A3 proteins has been shown to be important for substrate binding and recognition [41-46] (reviewed in refs. [21,47]) was also subjected to mutagenesis. Several of the mutant proteins e.g., W115A, W115F, del(115), and Y113D/H114D/W115Q (YHW/DDQ) were expressed at low levels compared to WT A3H (Figure 4D), possibly resulting from reduced protein stability due to removal of the large tryptophan side chain. Changing residues Y112, Y113, and H114, e.g., Y112A, Y113D/H114D (Figure 4F), and YHW/DDQ, which introduces polar residues from the A3G-CTD loop 7 (Figure 4E), led to the complete loss of deaminase activity. A similar result was obtained when W115 was deleted (del115) or changed to alanine (W115A) (Figure 4F). However, changing tryptophan to another aromatic residue, phenylalanine (W115F) (Figure 4F), led to only partial loss of deaminase activity, suggesting that these aromatic residues could be involved in base stacking interactions with the nucleic acids. Finally, we tested the deaminase activity of W115A, del115, Y113D/H114D, YHW/DDQ, and R110D at 10 μg total protein, but again, no activity was observed (data not shown). Collectively, these results suggest that the residues in A3H loop 7 are also likely to participate in specific interactions with nucleic acids.

### Role of A3H residues in A3H antiviral activity

Inhibition of HIV-1*vif* (−) replication by A3F and A3G is mediated by both deaminase-dependent and -independent mechanisms [20,61,65-79]. In an early study, it was concluded that A3H antiviral activity is deaminase-independent [80], but other reports indicated that this activity is dependent on catalysis [25,27]. It was therefore of interest to evaluate whether the presence or absence of deaminase activity (Figure 4) could be correlated with A3H antiviral activity (Figure 5) in our system.

To determine whether the inability of certain mutants to restrict HIV-1 replication was due to a defect in A3H packaging, Western blot analysis was performed (Figure 5A). Virions were probed for capsid protein (CA) as well as for A3H. For comparison, expression levels of A3H and tubulin in cells were also measured and the data were similar to the results in Figure 4A and D. Interestingly, several mutants that were expressed poorly in cells, packaged little or no A3H in virions (Figure 5A). These mutants include: R17D/R18D, Y113D/H114D, W115A, and del(154–157). Although W115F exhibited lower levels of protein than WT (Figures 4D and Figure 5A), a significant amount of A3H was encapsulated (Figure 5A).

Single-cycle infectivity assays of virions produced in cells expressing WT and mutant A3H proteins were performed using two different amounts of A3H plasmid (0.1 μg and 1 μg). Under conditions where the 0.1 μg dose was used, the antiviral activities of the mutants could be divided into three groups: (1) mutants that showed little or no deaminase or antiviral activities (i.e., having values similar to the empty vector control), such as R17D/R18D, R20D/R21D, del(20–22) (low level of deaminase activity), Y113D/H114D, W115A, W115F (reduced level of deaminase activity), del(115), and del(154–157) (Figure 5B); (2) mutants with WT levels of deaminase activity and appreciable antiviral activity, albeit lower than that of WT, such as T81S and V135I (Figure 5C); and (3) mutants completely lacking (P25R, E56A, T81A, Y112A) or having reduced levels (K168A/R171A) of deaminase activity that retain antiviral activity (Figure 5D).

The results obtained with mutants in groups 1 and 2 are consistent with deaminase-dependent antiviral activity, since group 1 mutants have neither activity and group 2 mutants have both. With the exception of R20D/R21D, del (20–22), and W115F, all of the group 1 mutants that were analyzed by Western blot exhibited packaging defects (see above), which would account for their lack of virion-associated deaminase and anti-HIV activities. Interestingly, the results with group 3 mutants were discordant and suggest that A3H may also utilize a deaminase-independent mechanism for HIV-1 restriction. Note that the levels of antiviral activity for these deaminase-negative mutants were still lower than WT values (0.1 μg condition), suggesting that deaminase activity is indeed required for maximal activity.

The antiviral activities of WT and a majority of the mutants increased upon increasing the transfected plasmid amount to 1 μg. Surprisingly, the antiviral activities of T81S and Y112A were similar to WT levels under this condition. The behavior of Y112A in our study differed from that of A3H Hap VII Y112A, which although expressed efficiently in cells, was poorly packaged and exhibited a very low level of anti-HIV-1 activity [29]. The explanation for this difference is not clear. We also performed a side-by-side comparison of the activities of the deaminase-negative catalytic mutants of A3G (E259Q) and A3H (E56A) as well as the respective WTs (Additional file 4: Figure S4) and found that both A3H and A3G WT and mutant samples displayed dose-dependent inhibition of HIV-1 infectivity. This suggests that A3G and A3H utilize a common mechanism for antiviral activity.

### Mechanism of A3H antiviral activity

A3G and A3F deaminase-independent anti-viral activity targets nascent DNA synthesis during reverse transcription [20,68-79]. To determine whether A3H deaminase-independent inhibition of HIV-1 infectivity is also associated with a reduction in viral DNA synthesis, we performed ERT assays using WT A3H and the active site mutant E56A.

**Figure 6:**
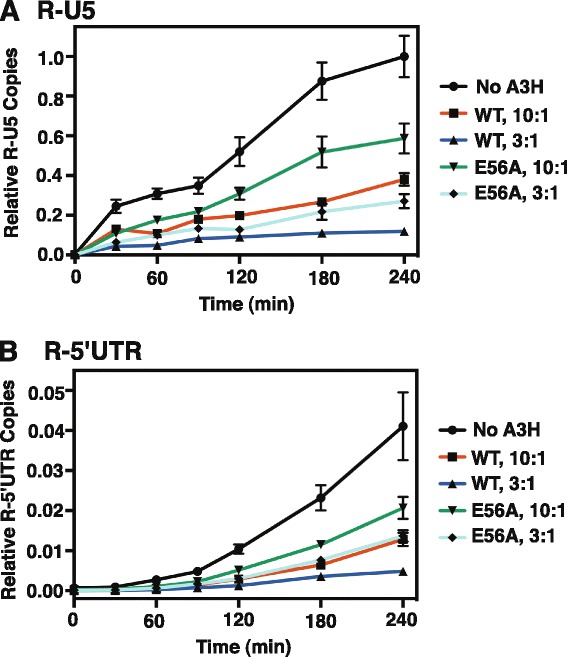
**ERT assays of virions produced following transfection of 293T cells with HIV-1** ***vif*** **(−) and WT A3H or the E56A active site mutant. (A and** **B)** Kinetics of DNA synthesis in ERT assays measuring the levels of R-U5 **(A)** and R-5′UTR **(B)**. The assays were performed as described in Methods. Note that synthesis of R-U5 DNA was more efficient than synthesis of R-5’UTR DNA in the presence of WT A3H under both the 10:1 and 3:1 conditions: the time required for 50% inhibition was 2 h for R-U5 and 3 h for R-5’UTR over the time course of the analysis.

In our assays, HIV-1*vif* (−) and A3H plasmids were transfected at two different ratios: 10:1 or 3:1, respectively (see Methods). Synthesis of R-U5 DNA (minus-strand strong-stop DNA) and R-5’UTR DNA (plus-strand DNA synthesized after plus-strand transfer) was measured over a 4-h time interval (Figure 6A and B). With WT A3H using the 10:1 condition, the levels of R-U5 were decreased to about 40% of the minus A3H control (100%) at 2 h (Figure 6A). The levels were drastically reduced for WT (3:1 condition) (~15% at 2 h) and the time course showed no appreciable change in level over the 4-h window. The E56A mutant, which lacks deaminase activity, was also capable of reducing the R-U5 levels to about 60% at 2 h (10:1 condition). At the higher dose (3:1 condition), R-U5 levels were further reduced relative to the control (~25% at 2 h), but the inhibition did not saturate even after 4 h, indicating partial inhibition. Similar trends for the WT and E56A mutant were also observed when synthesis of R-5′UTR (plus-strand synthesis) was monitored (Figure 6B). These results demonstrate that A3H deaminase-independent HIV-1 restriction involves inhibition of viral DNA synthesis.

## Discussion

In this work, we use sequence- and structure-guided mutagenesis to provide a detailed analysis of A3H deaminase activity and to correlate enzymatic function with antiviral activity. In addition, we identify the A3H structural elements associated with these activities. We also show that A3H deaminase activity in cell extracts is suppressed by binding to cellular RNAs (Figure 3), consistent with an earlier report indicating that A3H interacts strongly with 7SL, Y1, Y3, and Y4 RNAs in 293T cells [57]. Interestingly, extensive mutagenesis studies demonstrate that the determinants of deaminase and antiviral activities are not necessarily the same, as a number of deaminase-negative mutants retain antiviral activity (Figures 4 and 5). Thus, A3H appears to inhibit HIV-1 infectivity via deaminase-dependent and -independent mechanisms.

To assess the molecular and structural properties of A3H Hap II, we generated a homology model based on the crystal structure of A3G-CTD (PDB: 3IR2) [53]. The electrostatic surface potential of the A3H model identified a “large basic patch” (Figure 2B, blue region), containing a cluster of basic residues that could be potentially involved in specific binding to ssDNA substrates as well as binding to cellular RNAs. While this manuscript was under review, a paper by Shandilya et al. [81] appeared, presenting homology models of the individual domains of several A3 proteins, including A3H. An electrostatic map of this A3H model also revealed a large basic patch. In fact, comparison of the two structures did not show any significant structural differences.

The basic nature of A3H is important and impacts catalytic function. Thus, deaminase activity is strongly inhibited upon mutating a subset of the basic residues (R17, R18, R20, R21, Figure 4C; R110, Figure 4F; K168, R171, Figure 4E), suggesting reduced binding to the 40-nt nucleic acid substrate. Arginine mutants that were tested also lacked antiviral activity (Figure 5B). However, the basic residues may not all function in the same manner, since the double mutant K168A/R171A retained some deaminase (Figure 4E) as well as restriction activity (Figure 5D). Interestingly, the region corresponding to R17 to P22 in A3H (containing four basic residues) is part of an alpha helix (Figure 2), but is not conserved in other A3 domains (Figure 1). In A3A, this region is completely missing, while in A3C, A3G-CTD, and A3F-CTD, there is a loss of four (A3C, A3G-CTD) or two (A3F-CTD) basic residues. Deletion of A3H residues R20, R21, and P22 (the del(20–22) mutant) results in significant loss, but not complete abolition of deaminase activity (Figure 4C), possibly due to perturbation of the local structure in the mutant protein. Although this mutant is efficiently packaged into virions (Figure 5A), it cannot restrict HIV-1 (Figure 5B).

Residues in A3H that are unique to its Z3 domain e.g., T81 and V135 are replaced in other active deaminase domains by S and I respectively, which are chemically similar (Figure 1 and Additional file 1: Figure S1). This implies similarity in function and is consistent with our finding that the A3H mutants T81S and V135I, retain enzymatic (Figure 4B and E, respectively) as well as restriction activity (Figure 5C). However, the T81A mutant in which the hydroxyl group common to T and S, is removed, has only background levels of deaminase activity (Figure 4B). This suggests an important structural role for the hydroxyl group at this position, possibly participation in hydrogen bonding interactions with a nearby side chain. In fact, the Oγ atom of S284, the corresponding residue in A3G, is within hydrogen bonding distance to the Y219 H_N_ backbone atom and is also observed in other A3 proteins [41,43,49,54]. It is interesting that despite lacking enzymatic activity, the T81A mutant exhibits significant antiviral activity (Figure 5D).

Although the loop 7 element of A3 proteins plays an essential role in determining deaminase specificity, each protein possesses a unique sequence (Figure 1) and is affected differently when subjected to mutation. For example, as indicated by modeling the A3A/ssDNA complex, the A3A loop 7 cluster of residues (127-ARI**YDYD**PL-135) interacts with the T (D133) and A (D131, Y132, and D133) nucleotides in the T**C**A motif, while D131 makes contacts with the central C [41]. Substitutions in all four cluster residues lead to complete loss of A3A deaminase activity [42]. In the case of the A3G-CTD loop 7 (313-RI**YDDQ**GR-320), mutation of the only aromatic residue, Y315, negatively impacts deaminase activity [43]. Additionally, changing both D316 and D317 to R [43] or replacing D317 with the corresponding A3A residue (Y132) [46] results in altered deaminase substrate specificity, as manifested by preferential deamination of the central C in the motif C**C**C instead of the 3′ C (CC**C**). Exchanging the A3H loop 7 (109-SRL**YYHW**CK-118) cluster residues YHW with the corresponding A3G-CTD residues, DDQ (YYHW→YDDQ), abrogates deamination activity (Figure 4E). This could reflect a change in substrate specificity or in the details of the nucleic acid binding mode due to a protein conformational change. Interestingly, a similar switch in sequence in the case of A3A (YDYD→YDDD) also reduces activity to background levels [42].

The importance of the A3H W115 residue in loop 7 is underscored by our finding that mutation of W115 to A results in low cellular protein levels (Figures 4D and 5A), blocks packaging into virions (Figure 5A), and eliminates deaminase (Figure 4F) and antiviral (Figure 5B) activities. Wang et al. [29] were unable to detect expression of (Hap VII) W115A in 293T cells, while Zhen et al. [57] reported that (Hap II) W115A is present at low levels in cell lysates. However, even when expression is equivalent to that of WT (by transfecting cells with a high amount of the Hap II mutant plasmid), W115A is barely detectable in viral lysates, does not restrict HIV-1, and binds cellular RNAs with greatly reduced efficiency [57]. Collectively, these results lend further support to the conclusion that the identity of loop 7 residues is critical for enzyme function.

Surprisingly, although A3H is highly basic and has only one domain, it more closely resembles the double-domain proteins A3D, A3F, and A3G than the single-domain A3A and A3C proteins with respect to several important biological properties. For example, like A3D, A3F, and A3G [53,64,82-92], A3H associates in solution in the absence of nucleic acid [93] and forms multimers and high molecular weight ribonucleoprotein complexes in cells [92,94]. In addition, it restricts the infectivity of HIV-1*vif* (−) virions (Figure 5) [26,27,62,80,94]. The anti-HIV-1 activity of A3 proteins A3D, A3F, A3G, and A3H involves (i) packaging of the proteins into the cores of nascent virions in the producer cell [28,36,95,96]; and (ii) inhibition of viral replication in the target cell (reviewed in ref. [9]). These parameters are linked to subcellular localization, which varies among the A3 proteins. However, in this case too, just as A3D, A3F, and A3G are located in the cytoplasm [82-84,86,92,97-99], A3H haplotypes that have antiviral activity (e.g., Hap II) are predominantly cytoplasmic [28,100].

The packaging of antiviral A3 proteins inside the HIV-1 core allows them to interact directly with viral nucleic acids (genomic RNA and nascent DNA synthesized during reverse transcription) in the presence of nucleocapsid protein and reverse transcriptase (RT). Here we show that A3H inhibits viral infectivity by both deaminase-dependent and -independent mechanisms (Figure 5), although it is likely that deaminase-dependent activity is dominant (compare data for WT and the catalytic mutant E56A in Figure 5D, Figure 6, Additional file 4: Figure S4). For A3G, we have proposed a “roadblock” mechanism that is based on its tight nucleic acid binding [61,101] and that is independent of catalytic activity [69,74]. We reasoned that since A3G displays slow on-off binding kinetics [69,79], RT is unable to traverse the template when A3G is bound and consequently, RT-catalyzed polymerization is blocked. Recent single molecule stretching studies in support of this mechanism showed that A3G can transform from a fast enzyme (required for deamination) to a slow enzyme (causing a roadblock) during the course of protein oligomerization [79].

The highly basic character of the A3H protein (Figure 2B, Table 1) (like the NTDs of A3D, A3F, and A3G) strongly suggests that it also binds tightly to viral RNA and DNA, in agreement with studies of A3H binding to cellular RNA [57]. Moreover, the observed reduction in minus- and plus-strand DNA synthesis by RT, even in the absence of deaminase activity (Figure 6) and the fact that A3H can multimerize [92,93] suggest that a roadblock mechanism might also be relevant to A3H deaminase-independent HIV-1 restriction (Figure 6). Interestingly, although A3H mediates hypermutation of HIV-1 [27,48,62] and HBV [17] DNA, the antiviral activity of A3H against HTLV-1 does not involve editing [15].

In summary, A3H as well as A3D, A3F, and A3G constitute a group of A3 proteins used by the human innate immune system in its arsenal against HIV-1. The selective pressure to maintain expression of antiviral haplotypes of A3H in certain populations warrants greater understanding of this protein in terms of its molecular properties. Here, we present such an analysis and correlate enzyme function and antiviral activity. Although A3H is a single-domain protein and the most divergent member of the A3 family, we show that it utilizes strategies similar to those used by other antiviral double-domain A3 proteins (A3D, A3F, and A3G) to counteract HIV-1 infectivity. Knowledge of A3H structure-function relationships should be invaluable for the design of drugs to modulate A3H deaminase activity and augment its anti-HIV effect. Furthermore, since A3H is a single-domain protein and contains a unique Z3 domain, it may present a more specific target than the double-domain antiviral A3 proteins. Thus, taken together, A3H clearly provides a molecular paradigm to explore the antiviral response of A3 proteins to retroviral pathogens.

## Conclusions

Based on a homology model of A3H Hap II and the results of extensive mutagenesis, we have identified structural elements and key residues associated with A3H deaminase and anti-HIV-1 activities. In addition, we provide evidence that A3H restriction of HIV-1 replication and inhibition of reverse transcription occur by deaminase-dependent and -independent mechanisms.

## Methods

### Materials

DNA oligonucleotides labeled with AlexaFluor 488® were obtained from Integrated DNA Technologies (Coralville, IA). The concentration of each oligonucleotide was determined by measuring its absorbance at 260 nm, using the extinction coefficients provided by the manufacturer. RNase A (endonuclease-free) was purchased from Qiagen Inc. (Germantown, MD). *Escherichia coli* uracil DNA glycosylase (UDG) was obtained from New England Biolabs (Beverly, MA). Gel loading buffer and nuclease-free water were purchased from Ambion® (Life Technologies, Grand Island, NY). Anti-A3H sera (p3A3 or p1H6), anti-HIV-1 CA sera (for ERT experiments), and TZM-bl cells (from John C. Kappes, Xiaoyun Wu, and Tranzyme Inc.) [102-104] were obtained from the AIDS Research and Reference Reagent Program (Division of AIDS, NIAID, NIH). An anti-HIV-1 p24 monoclonal antibody was purchased from ZeptoMetrix (Franklin, MA) and was used for detection of CA and Pr55^*gag*^ in virions and cell lysates, respectively (see Figure 5). A monoclonal antibody (Anti-FLAG M2) against a FLAG tag was obtained from Sigma-Aldrich (St. Louis, MO). Anti-tubulin antibody was purchased from Abcam (Cambridge, MA).

### Construction of the A3H HapII homology model

The initial A3H Hap II homology model was constructed without a Zn atom and was based on the A3G-CTD crystal structure (PDB: 3IR2) [53] by using MODELLER version 9v8 [56] and the sequence alignment shown in Figure 1. The Zn atom was subsequently added to the structure at a position equivalent to that in the structure of A3G-CTD. This was followed by energy minimization where only the Zn ion and the H54, C85, C88 side-chains were allowed to move. During the energy minimization, the lengths of the coordination bonds were kept as follows: 1.90 Å for the bond between the Zn ion and the H54 N_δ1_ atom; and 2.25 Å for the bond between the Zn ion and the C85 or C88 S_γ_ atom. The charge of individual atoms and their radius parameters based on an amber force field [105] were generated by the pdb2pqr program [106]. Partial charges of atoms within the Zn coordination site were manually adjusted to +1e for the Zn ion, −0.6e for the S_γ_ atom and +0.1e for the C_β_ atom of the cysteines to account for the approximate redistribution of the charges between the two reduced cysteines and Zn ion through the coordination bonds. All structure and electrostatic potential map figures were generated with MOLMOL [107]. Sequence identity was determined with DNAStar (http://www.dnastar.com/megalign_help/index.html#!Documents/calculationofpercent.htm).

### Construction of A3H mutants

pTR600 mammalian expression plasmids without an insert [108] or containing the A3H HapII coding sequence [27] were a generous gift from Viviana Simon (Mount Sinai School of Medicine, New York, NY) and had either no tag or an N-terminal Flag tag. A3H mutants were all constructed in the pTR600 A3H-Flag vector using the QuikChange Lightning Site-Directed Mutagenesis Kit (Agilent Technologies, Santa Clara, CA) or the Tagmaster Site-Directed Mutagenesis Kit (GM Biosciences, Rockville, MD). For primer design, the on-line QuikChange Primer Design Program provided by the manufacturer or the design guidelines provided with the Tagmaster kit were used. Large-scale plasmid preparations were obtained using the HiSpeed Plasmid Maxi Prep kit (Qiagen, Inc.). The A3H sequence in each plasmid was verified by DNA sequencing performed by ACGT (Wheeling, IL).

### Preparation of mammalian cell extracts

Propagation of 293T cells, transfection procedure, preparation of cell extracts, and determination of protein concentration were performed as detailed in Mitra et al. [42]. The expression levels of A3H were estimated by subjecting ~20 μg of total protein in the extract to Western blot analysis using the Western Breeze chemiluminescent Western blot kit (Life Technologies). The primary antibodies used for this analysis were anti-A3H or anti-tubulin (loading control).

### Deaminase assay

Prior to performing the deaminase assay, the 293T cell extract (20 μg) was treated with RNase A (Qiagen, final concentration 1 μg/μl) in a 20-μl reaction volume and incubated at 37°C for 15 min, unless indicated otherwise. Details of the deaminase assays and polyacrylamide gel analysis are described in Mitra et al. [42]. A list of oligonucleotides used for the deaminase assays is given in Additional file 5: Table S1. Throughout the text, the dC residue that is deaminated is highlighted in bold (C). A 40-nt Alexa-Fluor 488-labeled ssDNA (JL913) containing the TT**C**A deaminase motif was used as the substrate, unless specified otherwise. To calculate the percent deaminase product, the product signal intensity for each lane was divided by total signal intensity and multiplied by 100. The data presented represent the average of two determinations from two independent transfections. Note that the FLAG tag did not interfere with deaminase activity, as untagged and tagged WT A3H showed similar levels of activity (Figure 4C).

### HIV-1 infectivity assay

To assay the effect of expressing A3H WT or mutant proteins on HIV-1 infectivity, 293T cells were cotransfected with 1.0 μg of pNL4–3*vif* (−), which was kindly provided by Klaus Strebel (National Institute of Allergy and Infectious Diseases, National Institutes of Health, Bethesda, MD) and 1.0 or 0.1 μg of the pTR600 A3H HapII plasmid (WT or mutant) or pTR600 empty vector (control), using the FuGENE HD transfection agent (Promega, Madison, WI) as previously described [49]. Virus-containing supernatants were collected 48 h after transfection and filtered. The amount of CA protein (i.e., p24 antigen) in the supernatant was determined by ELISA assay (ZeptoMetrix, Buffalo, NY). TZM-bl indicator cells were infected with viral supernatant containing 10 ng of CA. Relative infectivity expressed as relative light units (RLU) was measured using the Bright-Glo luciferase assay system kit (Promega) and an ARVO MX luminescence counter (PerkinElmer, Waltham, MA). The data represent the results of three independent experiments.

### ERT assay

The following plasmids were used: pTR600, empty vector; WT A3H HapII expressed as an N-terminal FLAG-tagged protein in pTR600 (pA3H WT); A3H E56A active site mutant expressed as an N-terminal FLAG-tagged protein in pTR600 (pA3H E56A); and pNL4-3*vif* (−).

Transfections were performed as described previously [109], except that TransIT-293 from Mirus Bio LLC (Madison, WI) was used, according to the manufacturer’s instructions. Briefly, 100-mm cell culture dishes were seeded with 3 × 10^5^ 293T cells in Dulbecco’s modified Eagle’s Medium with 10% fetal bovine serum. Two days later, the cells were transfected in duplicate with 15 μg of pNL4-3*vif* (−) and 0, 1.5, or 5 μg of pA3H WT or pA3H E56A plasmid; the molar ratios of pNL4-3*vif*(−) to A3H plasmid were therefore 10:1 or 3:1, respectively. The pTR600 plasmid was the negative control and was also used to generate a constant level (5 μg) of transfected pTR600 DNA, with or without the A3H insert. Culture fluids were changed 24 h after transfection, then harvested after two consecutive 24-h periods, and passed through 0.22 μm filters. Samples from each culture were pooled and were treated sequentially with DNase I and subtilisin, as described [109,110].

ERT reactions were performed without Triton X-100 pretreatment and nucleic acids were isolated as detailed in Thomas et al. [109]. Real-time PCR for the detection of R-U5 and R-5’UTR was performed in duplicate as described previously [111]. The kinetics of DNA synthesis were plotted and the data were normalized relative to maximal R-U5 copies at 240 min for pNL4-3*vif* (−) in the absence of A3H cotransfected plasmid. Each data point was the result of two independent transfections, each measured in duplicate. Error bars represent the standard error of the mean.

## Additional files

Additional file 1: Figure S1. Sequence alignment of residues in the Zn-binding (Z) domains of the seven A3 proteins (A to H). The Zn-coordinating (H and C) and active site (E) residues are highlighted in light blue. A3H residues that differ from highly conserved residues at the corresponding positions in the other A3 proteins are shown in lavender. The residues in loop 7 are bracketed. The numbers at the end of each line represent the position in the full-length protein. The percent sequence identity of each Z domain relative to that of A3H (defined as 100%) is indicated. The sequence alignment was performed using Lasergene software (DNASTAR, Inc., Madison, WI). Additional file 2: Figure S2. Superposition of the structures of the current A3H model and other A3 proteins in ribbon representation. The backbone traces are colored gray (A3H) and green (other A3 proteins). The Zn ions are shown in brown (A3H) and in coral (other A3 proteins). **(A)** A3H and A3G-CTD. **(B)** A3H and A3A. **(C)** A3H and A3C. **(D)** A3H and A3F-CTD. Additional file 3: Figure S3. Electrostatic surface potential maps of A3H and other A3 proteins. **(A)** A3H. **(B)** A3G-CTD. **(C)** A3A. **(D)** A3C. **(E)** A3F-CTD. Regions with positive and negative electrostatic potentials are highlighted in blue and red, respectively. Additional file 4: Figure S4. Comparison of antiviral activities of A3H and A3G WT and catalytic mutants. Virions produced from 293T cells transfected with HIV-1*vif*(−) (1 μg) and either 0.1 (gray bars) or 1 μg (black bars) of the indicated A3H or A3G WT or mutant plasmids. Infectivity was assayed as described in Methods. The catalytic mutants are E56A (A3H) and E259Q (A3G). A vector control (100% infectivity) was also included. Additional file 5: Table S1. Oligonucleotides used for deaminase assays.

## Abbreviations

A3: APOBEC3
ss: Single-stranded
HIV-1: Human immunodeficiency virus type 1
HTLV-1: Human T-lymphotropic virus type 1
HBV: Hepatitis B virus
CTD: C-terminal domain
NTD: N-terminal domain
WT: Wild type
ERT: Endogenous reverse transcription
CA: Capsid protein
RT: Reverse transcriptase
