# Supplementary material for: Sequence and structural determinants of human APOBEC3H deaminase and anti-HIV-1 activities
Source: Retrovirology. 2015 Jan 22;12:3. doi: 10.1186/s12977-014-0130-8 (PMC4323217; doi:10.1186/s12977-014-0130-8)
Supplement: Additional file 1: Figure S1. — Sequence alignment of residues in the Zn-binding (Z) domains of the seven A3 proteins (A to H). The Zn-coordinating (H and C) and active site (E) residues are highlighted in light blue. A3H residues that differ from highly conserved residues at the corresponding positions in the other A3 proteins are shown in lavender. The residues in loop 7 are bracketed. The numbers at the end of each line represent the position in the full-length protein. The percent sequence identity of each Z domain relative to that of A3H (defined as 100%) is indicated. The sequence alignment was performed using Lasergene software (DNASTAR, Inc., Madison, WI). [file 12977_2014_130_MOESM1_ESM.pdf]

Figure S1

```
A3A (Z1)     AKNLLCGFYGRHAELRFLDLVPS-LQLDPAQIYRVTWFISWSPCFSWGCAGEVRAFLQENTHVRLRIFAA 127
A3B-NTD (Z2) ----VYFKPQYHAEMCFLSWFCG-NQLPAYKCFQITWFVSWTPCPD--CVAKLAEFLSEHPNVTLTISAA 121
A3B-CTD (Z1) AKNLLCGFYGRHAELRFLDLVPS-LQLDPAQIYRVTWFISWSPCFSWGCAGEVRAFLQENTHVRLRIFAA 310
A3C (Z2)     ----VDSETHCHAERCFLSWFCD-DILSPNTKYQVTWYTSWSPCPD--CAGEVAEFLARHSNVNLTIFTA 121
A3D-NTD (Z2) ----VYFRFENHAEMCFLSWFCG-NRLPANRRFQITWFVSWNPCLP--CVVKVTKFLAEHPNVTLTISAA 133
A3D-CTD (Z2) ----VDPETHCHAERCFLSWFCD-DILSPNTNYEVTWYTSWSPCPE--CAGEVAEFLARHSNVNLTIFTA 317
A3F-NTD (Z2) ----VYSQPEHHAEMCFLSWFCG-NQLPAYKCFQITWFVSWTPCPD--CVAKLAEFLAEHPNVTLTISAA 119
A3F-CTD (Z2) ----VDPETHCHAERCFLSWFCD-DILSPNTNYEVTWYTSWSPCPE--CAGEVAEFLARHSNVNLTIFTA 304
A3G-NTD (Z2) ----VYSELKYHPEMRFFHWFSKWRKLHRDQEYEVTWYISWSPCTK--CTRDMATFLAEDPKVTLTIFVA 121
A3G-CTD (Z1) APHKHGFLEGRHAELCFLDVIPF-WKLDLDQDYRVTCFTSWSPCFS--CAQEMAKFISKNKHVSLCIFTA 312
A3H (Z3)     --------KKCHAEICFINEIKS-MGLDETQCYQVTCYLTWSPCSS--CAWELVDFIKAHDHLNLRIFAS 109
```

```
             Loop 7
                                                  % Identity
A3A (Z1)     RIYDY-DPLYKEALQMLRDAG----AQVSIMTYD 156     36
A3B-NTD (Z2) RLYYYWERDYRRALCRLSQAG----ARVTIMDYE 151     31
A3B-CTD (Z1) RIYDY-DPLYKEALQMLRDAG----AQVSIMTYD 339     36
A3C (Z2)     RLYYFQYPCYQEGLRSLSQEG----VAVEIMDYE 151     43
A3D-NTD (Z2) RLYYYRDRDWRWVLLRLHKAG----ARVKIMDYE 163     28
A3D-CTD (Z2) RLCYFWDTDYQEGLCSLSQEG----ASVKIMGYK 347     39
A3F-NTD (Z2) RLYYYWERDYRRALCRLSQAG----ARVKIMDDE 150     31
A3F-CTD (Z2) RLYYFWDTDYQEGLRSLSQEG----ASVEIMGYK 334     43
A3G-NTD (Z2) RLYYFWDPDYQEALRSLCQKRDGPRATMKIMNYD 155     34
A3G-CTD (Z1) RIYDD-QGRCQEGLRTLAEAG----AKISIMTYS 341     40
A3H (Z3)     RLYYHWCKPQQDGLRLLCGSQ----VPVEVMGFP 139    100
```
