# Supplementary figures and images for: Sequence and structural determinants of human APOBEC3H deaminase and anti-HIV-1 activities
Source: Retrovirology. 2015 Jan 22;12:3. doi: 10.1186/s12977-014-0130-8 (PMC4323217; doi:10.1186/s12977-014-0130-8)

**Figure S2**

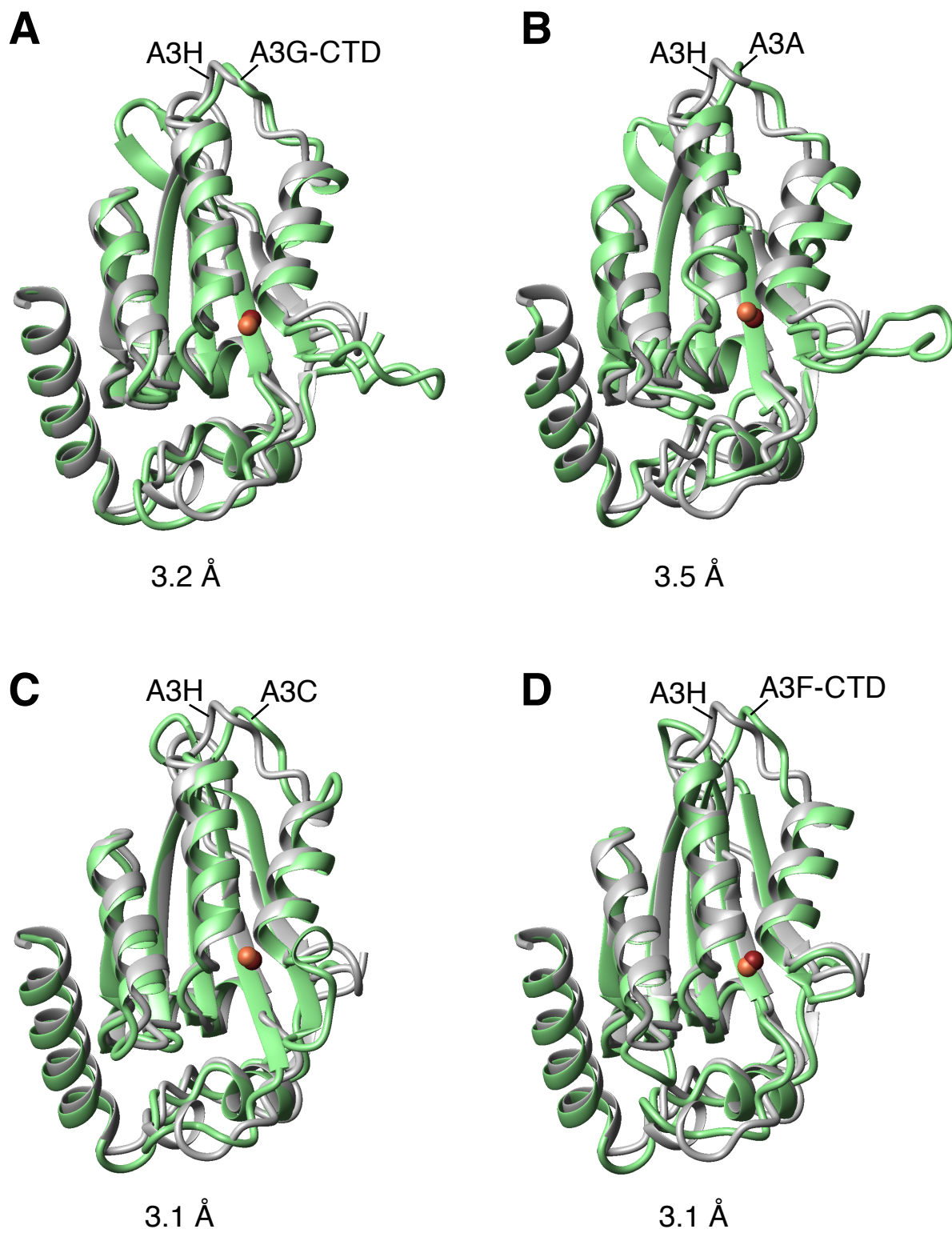

Supplement: Additional file 2: Figure S2. — Superposition of the structures of the current A3H model and other A3 proteins in ribbon representation. The backbone traces are colored gray (A3H) and green (other A3 proteins). The Zn ions are shown in brown (A3H) and in coral (other A3 proteins). (A) A3H and A3G-CTD. (B) A3H and A3A. (C) A3H and A3C. (D) A3H and A3F-CTD. [file 12977_2014_130_MOESM2_ESM.pdf]

**Figure S3**

**A** A3H

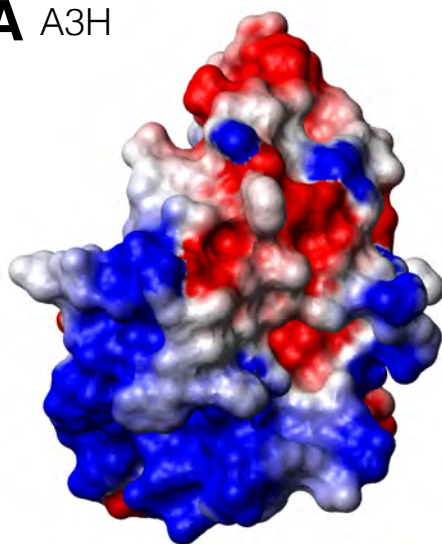

**B** A3G-CTD

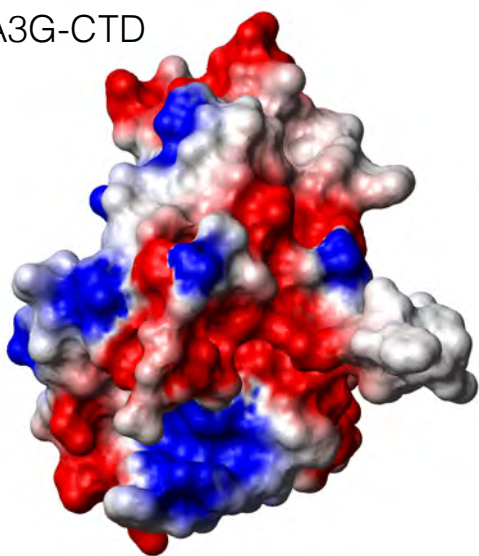

**C** A3A

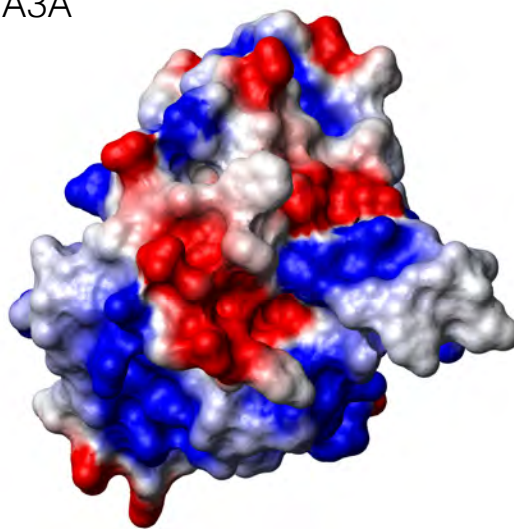

**D** A3C

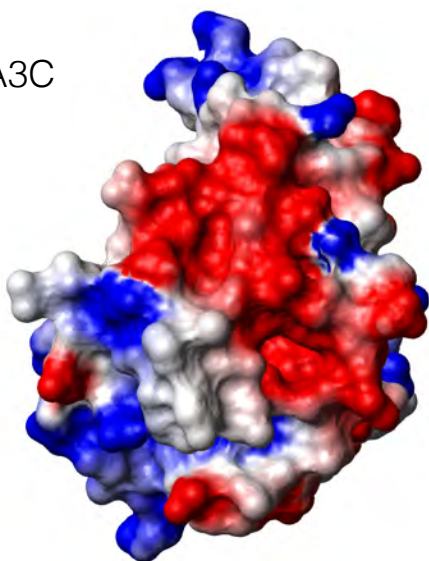

**E** A3F-CTD

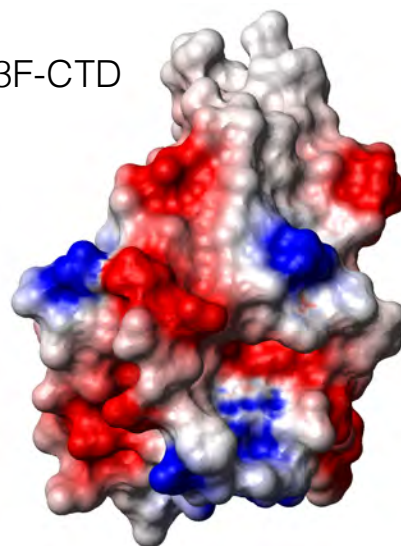

Supplement: Additional file 3: Figure S3. — Electrostatic surface potential maps of A3H and other A3 proteins. (A) A3H. (B) A3G-CTD. (C) A3A. (D) A3C. (E) A3F-CTD. Regions with positive and negative electrostatic potentials are highlighted in blue and red, respectively. [file 12977_2014_130_MOESM3_ESM.pdf]

Figure S4

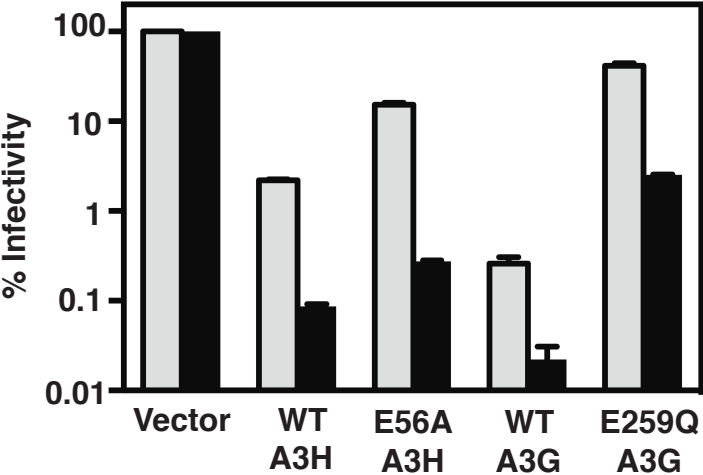

Supplement: Additional file 4: Figure S4. — Comparison of antiviral activities of A3H and A3G WT and catalytic mutants. Virions produced from 293T cells transfected with HIV-1vif(−) (1 μg) and either 0.1 (gray bars) or 1 μg (black bars) of the indicated A3H or A3G WT or mutant plasmids. Infectivity was assayed as described in Methods. The catalytic mutants are E56A (A3H) and E259Q (A3G). A vector control (100% infectivity) was also included. [file 12977_2014_130_MOESM4_ESM.pdf]
