# Supplementary material for: Sequence and structural determinants of human APOBEC3H deaminase and anti-HIV-1 activities
Source: Retrovirology. 2015 Jan 22;12:3. doi: 10.1186/s12977-014-0130-8 (PMC4323217; doi:10.1186/s12977-014-0130-8)
Supplement: Additional file 5: Table S1. — Oligonucleotides used for deaminase assays. [file 12977_2014_130_MOESM5_ESM.pdf]

Additonal File 5

Table S1 Oligonucleotides used for deaminase assays

Name	Length (nt)	Sequence
**JL-913**	40	5'- (Alexa488) ATT ATT ATT ATT ATT ATT ATT TCA TTT ATT TAT TTA TTT A -3'
**JL-1043**	40	5'- (Alexa488) ATT ATT ATT ATT ATT ATT ATT TCT TTT ATT TAT TTA TTT A -3'
**JL-1094**	40	5'- (Alexa488) ATT ATT ATT ATT ATT ATT ATT TCG TTT ATT TAT TTA TTT A -3'
**JL-1095**	40	5'- (Alexa488) ATT ATT ATT ATT ATT ATT ATA CCC ATT ATT TAT TTA TTT A -3'
**JL-1178**	40	5'- (Alexa488) ATT ATT ATT ATT ATT ATT ATT GCA TTT ATT TAT TTA TTT A -3'
